# Morphogenetic Identification of a New Record *Condica capensis* (Lepidoptera: Noctuidae) in Yunnan, China

**DOI:** 10.3390/insects16020130

**Published:** 2025-01-29

**Authors:** Pengfan Qian, Jiayin Fan, Xiaoyuan Zhang, Minfang Zeng, Xiaolong Han, Yonghe Li, Xulu Luo

**Affiliations:** 1 College of Plant Protection, Yunnan Agricultural University, Kunming 650201, China; 2023210338@stu.ynau.edu.cn (P.Q.); 15996142969@163.com (J.F.); 2020210320@stu.ynau.edu.cn (X.Z.); 2023240467@stu.ynau.edu.cn (M.Z.); 2 Office of Student Affairs, Yunnan Agricultural University, Kunming 650201, China; hanxiaolong@ynau.edu.cn; 3 College of Landscape and Horticulture, Yunnan Agricultural University, Kunming 650201, China

**Keywords:** *Carthamus tinctorius*, COI, *Condica capensis*, DNA barcode, insect identification, morphology, safflower

## Abstract

*Condica capensis* (Lepidoptera: Noctuidae), a newly identified pest in Yunnan Province, China, poses a threat to safflower crops. Discovered in Nanhua County in November 2023, the pest damages safflower at multiple life stages, especially during its larval stage, when it feeds on leaves, tender stems, and flower filaments, sometimes causing the entire plant to die. Morphological and molecular analyses, including mitochondrial *cytochrome C oxidase I* (COI) gene sequencing, confirmed its identity as *C. capensis*, a new species record for Yunnan. The study also documented the pest’s life cycle, reproductive behavior, and natural enemies, highlighting the potential for biological control using parasitic wasps such as *Cotesia* sp. This research emphasizes the need for accurate pest identification and monitoring to develop effective, sustainable pest management strategies. As safflower cultivation grows in Yunnan, managing *C. capensis* is critical to safeguarding local agriculture and preventing broader agricultural threats.

## Simple Summary

Safflower is a valuable crop used in various industries, but it suffers from pest attacks. Recently, *Condica capensis*, a pest previously unreported in China, was found in Yunnan Province. Our study examines its physical and biological traits, life cycle, and natural enemies. By identifying and understanding this pest, we aim to help farmers and researchers effectively manage and control its spread, ensuring healthy safflower cultivation.

## 1. Introduction

*Carthamus tinctorius,* commonly known as safflower, is an annual herb from the genus *Carthamus* in the Asteraceae family [1]. This plant is valued for its medicinal properties, with modern research highlighting its broad biological activities, including immune modulation, anti-inflammatory, antioxidant, anti-aging, anti-fatigue, anti-tumor, and analgesic effects [2]. Historically, safflower has been used for over 4000 years, serving as a primary source of red–yellow dye in ancient Egypt [3] and one of the most famous natural red dyes for silk during the Qing Dynasty in China [4]. It is also cultivated extensively in North America and India for its seeds, which are used as an oilseed crop [5,6]. Additionally, safflower oil is utilized in various industries, including food, cosmetics, pharmaceuticals, and biofuels, making it a highly valuable plant resource [7].

Originally native to the Near East [8], safflower is now cultivated in over 60 countries worldwide [9]. However, as the global cultivation of safflower expands, pest infestations have emerged as a significant threat to the industry. In Iran, major pests affecting safflower include *Acanthiophilus helianthi*, *Autographa gamma*, and *Helicoverpa armigera* [10]. In Brazil, *Euphoria lurida* has been reported feeding on safflower flower buds [11]. In Turkey, Yucel identified eight species of aphids, including *Aphis craccivora*, *A. fabae*, *Brachycaudus cardui*, *B. helichrysi*, *Myzus persicae*, *Uroleucon aeneum*, *U. carthami*, and *U. jaceae*, that infest safflower [12]. Although safflower has a long cultivation history in China, spanning over 2000 years, reports of safflower pests remain scarce. In November 2023, our research team identified a new pest in Nanhua County, Yunnan Province, China, infesting safflower leaves. Morphological and molecular analysis confirmed the pest to be *Condica capensis*, a lepidopteran species. This represents a new record for southwestern China, as no prior reports have documented *C. capensis* as a pest of safflower in this region.

First discovered at Cape of Good Hope, Africa in 1852 and originally named *Apamea apensis* Guenée, the species was later reclassified to *Perigea capensis* in 1908 and eventually re-named *Condica. capensis* [13]. *C. capensis* is a polyphagous pest, feeding on a variety of plants, including species from the Acanthaceae family, such as *Acanthus* sp., and from the Asteraceae family, such as *Bidens pilosa* [14]. In Egypt, the species has been recorded feeding on a variety of Asteraceae plants, including *Ageratum* sp., *Calendula* sp., and *Cynara* sp., as well as plants from the *Macadamia* sp., and various grasses [15]. In India, it has been reported feeding on economic crops such as *Helianthus annuus* (sunflower), safflower, and *Gossypium hirsutum* (cotton) [16,17,18]. Recently, Giuseppe reported *C. capensis* as a new record from southern Italy, suggesting the possibility of its migration from North Africa through wind dispersal [14].

In India, Balikai documented the presence of *C. capensis* in safflower fields but did not investigate the morphological characteristics or biological traits of its different life stages, nor did they provide photographs for field identification [17]. This lack of detailed information has made it difficult to accurately identify *C. capensis* during field infestations. Thus, this study aims to investigate the morphology, biological characteristics, and natural enemies of *C. capensis* in Yunnan, providing a scientific foundation for its rapid and accurate identification, monitoring, and integrated pest management strategies.

## 2. Materials and Methods

### 2.1. Insect Collection

The experimental insects were collected from safflower fields in Xinhua Village, Nanhua County, Chuxiong Prefecture, Yunnan Province, China (latitude 25.32° N, longitude 101.15° E, altitude 1998.20 m, average annual temperature 15.7 °C, annual precipitation 795.1 mm) in November 2023.

### 2.2. Morphological Observation

The larvae were collected from infected safflower plants and transported to the laboratory, where they were fed fresh safflower leaves. The larvae were reared under controlled conditions (25 ± 1 °C, 65 ± 5% RH, 12L:12D light cycle). Morphological observations and measurements were conducted on each life stage (larvae, female and male pupae, female and male adults, and fertilized and unfertilized eggs) using a Leica M205 FA stereo microscope (Leica, Weztlar, Germany). For each life stage, 60 specimens were observed. Male genital dissections were performed following the method described by Chen et al. [19] with slight modifications. The male abdomen was removed, and the genitalia were dissected and placed in a 10% sodium hydroxide solution at 45 °C for 4–6 h (longer for larger specimens) to fully dissolve and remove other tissues. After treatment, the specimens were washed with distilled water and photographed under a stereo microscope.

### 2.3. Molecular Characterization of Condica capensis

#### 2.3.1. Genomic DNA Extraction

Genomic DNA was extracted from three larvae of *C. capensis* using the Animal Genomic DNA Extraction Kit (TSINGKE TSP202-200, Beijing, China). After homogenizing the larvae in a sterile tube, DNA extraction was performed according to the manufacturer’s protocol. The *cytochrome C oxidase I* (COI) gene was amplified using the primers LCO1490 (5′-GGTCAACAAATCATAAAGATATTGG-3′) and HCO2198 (5′-TAAACTT CAGGGTGACCAAAAAATCA-3′) [20].

#### 2.3.2. PCR Conditions

The PCR reaction mixture consisted of 45 μL of the enzyme mix (1× GoldenStar^®^ T6 Super PCR Mix Ver.2), 2 μL of forward primer (10 pmol), 2 μL of reverse primer (10 pmol), and 1 μL of DNA template, for a total volume of 50 μL. The PCR cycling conditions were as follows: initial denaturation at 94 °C for 2 min; followed by 5 cycles of denaturation at 94 °C for 30 s, annealing at 50 °C for 40 s, and extension at 72 °C for 1 min; then 35 cycles of denaturation at 94 °C for 30 s, annealing at 55 °C for 40 s, and extension at 72 °C for 1 min; and a final extension at 72 °C for 10 min. The reaction was stored at 4 °C.

#### 2.3.3. Nucleotide Sequence and Phylogenetic Analysis

The PCR products were verified by electrophoresis and bi-directionally sequenced by TSINGKE Biological Technology Co., Ltd. (Beijing, China). The sequencing results were compared for homology using BLAST against sequences in GenBank. Sequences with high similarity, as well as those from related genera, were downloaded. Phylogenetic trees were constructed using MEGA11 software [21], with *Chilo suppressalis* as the outgroup, employing the Neighbor-Joining (NJ) method [22].

#### 2.3.4. GenBank Accession Number

The COI sequence of *Condica capensis* reported in this study has been uploaded in GenBank (http://www.ncbi.nlm.nih.gov, accessed on 18 November 2024) under the accession number PQ613989.1.

### 2.4. Biological Studies

#### 2.4.1. Biology of *Condica capensis*

Mating trials were conducted with one male and one female per pair, selected from newly emerged adults (1:1 ratio), and placed in cylindrical PVC containers (bottom diameter: 4.5 cm, height: 17 cm) within a controlled artificial climate chamber (25 ± 1 °C, 65 ± 5% RH, 14L:10D light cycle). Mating and oviposition behaviors were observed for 30 pairs. The egg hatch duration, developmental stages of the larvae, pupation period, and adult longevity were recorded. The Brooks index and Crosby index were calculated according to the method by Loerch and Cameron [23]. Eggs were collected and measured under a stereo microscope (Leica, M205 FA, Weztlar, Germany) using Leica Application Suite X software to determine their height and diameter. For each larval instar, head capsule width, body length, and body width were measured using a stereo microscope. Newly formed male and female pupae were selected, and their length, width, and weight were measured using the same equipment, with weight determined using a precision electronic balance (Dibal, FA1004, Yancheng, China). Emerged male and female adults were collected and preserved in 50 mL plastic centrifuge tubes with cotton soaked in a suitable amount of ethyl acetate to immobilize the insects. Once dead, the adults were mounted on spreading boards to flatten their wings for morphological examination. Their wingspan, pronotum width, and body length were measured using a vernier caliper (Greener, 034180, Yantai, China). All measurements were based on 60 individuals (*n* = 60) under rearing conditions of 25 ± 1 °C, 65 ± 5% RH, and a 12L:12D light cycle.

#### 2.4.2. Natural Enemies of *Condica capensis*

Parasitoids naturally infesting *C. capensis* were collected and preserved in 75% alcohol. The specimens were sent to Professor Wang Xiaoyi at the Ecology and Nature Conservation Institute, Chinese Academy of Forestry, for identification.

### 2.5. Data Processing and Statistics

Data were statistically analyzed using EXCEL 2019 and visualized with R software (version 4.3.2) and associated packages (“gghalves”, “ggsignif”, “ggsci”, “ggpubr”, “tidyverse”, “agricolae”, “ggtrendline”, “ggplot2”). MEGA11 software was used to construct phylogenetic trees and analyze genetic distance.

## 3. Results

### 3.1. Morphological Redescription

#### 3.1.1. Egg

**Figure 1:**
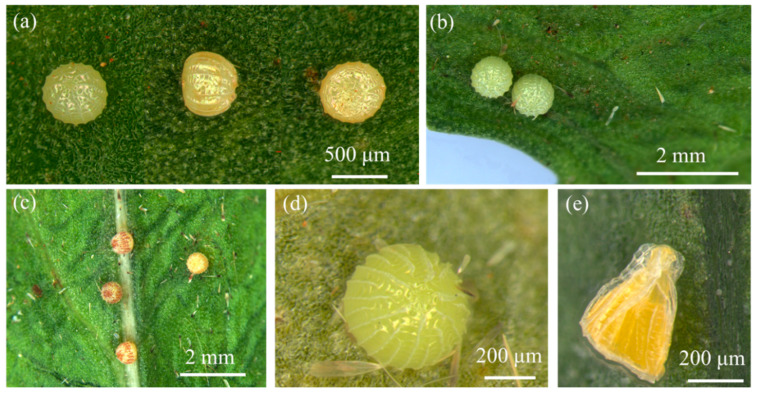
Morphological characteristics of *Condica capensis* eggs. (**a**) Dorsal, lateral, and ventral views of the egg; (**b**) freshly laid fertilized eggs; (**c**) fertilized eggs after 2–3 days; (**d**) freshly laid unfertilized egg; (**e**) unfertilized egg after 5–7 days.

The eggs are dome-shaped hemispheres. The micropyle is located at the center of the egg’s apex, surrounded by longitudinal ridges that radiate outward. These longitudinal ridges are interspersed with short, continuous horizontal ridges, forming a rectangular grid pattern (Figure 1a). Freshly laid fertilized eggs are pale yellow (Figure 1b). After 2–3 days, purple–brown spots appear on the egg surface, and the eggs turn purplish gray just before hatching (Figure 1c). Unfertilized eggs are initially pale yellow (Figure 1d) but begin to collapse after about a week under artificial conditions, although their color remains largely unchanged (Figure 1e).

#### 3.1.2. Larva

**Figure 2:**
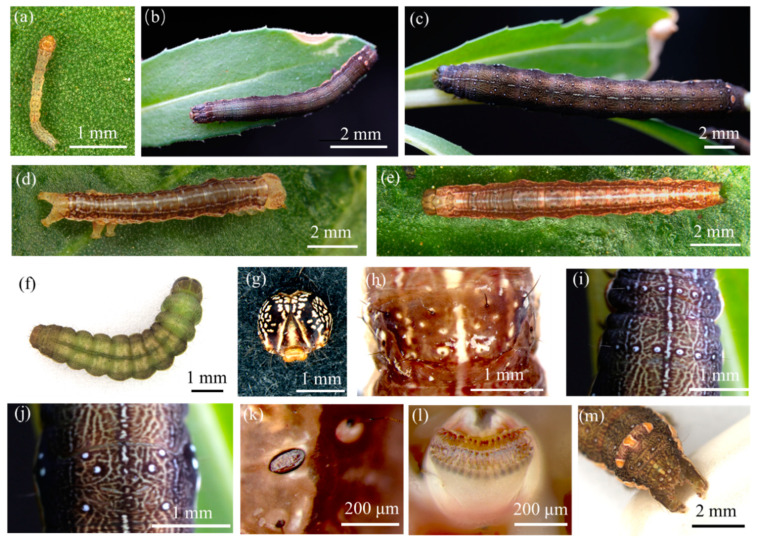
Morphological characteristics of *Condica capensis* larvae. (**a**) 1st instar larva; (**b**) 3rd instar larva; (**c**) 6th instar larva; (**d**) newly molted 2nd instar larva; (**e**) 2nd instar larva about to molt; (**f**) mature larva; (**g**) head; (**h**) prothoracic shield; (**i**) mesothorax and metathorax; (**j**) the first abdominal segment; (**k**) spiracle; (**l**) crochets; (**m**) anal plate.

The eruciform larvae exhibit significant color changes throughout development. Newly hatched larvae are pale yellow (Figure 2a). After the second instar, larvae are predominantly black (Figure 2b,c), though their bodies often turn dark brown just before and after molting (Figure 2d,e). Mature larvae initially turn dark brown and gradually change to green (Figure 2f). A few individuals remain green from the third instar until pupation. Head: Newly hatched larvae have yellow–brown genae. As the larvae grow, the genae turn black, with irregular white spots on both sides. The frons of young larvae is yellowish white, turning orange in mature larvae. The adfrontal sclerites are yellowish white with black or dark brown edges. Both the clypeus and labrum are yellowish white, and the antennae are pale yellow (Figure 2g). Thorax: The prothorax has a pair of oval spiracles on each side. From a dorsal view, the prothoracic shield appears trapezoidal. In younger larvae, four black verrucae, each bearing setae, are visible on the upper and lower sides of the prothoracic shield. Additionally, two pairs of small, near-circular white spots along the dorsal midline, each with verrucae, are present (Figure 2h). The mesothorax and metathorax each have three pairs of black verrucae, arranged nearly in a straight line. In older larvae, near-circular white spots around the verrucae become more prominent (Figure 2i). There are three pairs of thoracic legs. Abdomen: From the dorsal view, each abdominal segment has three pairs of verrucae arranged in an “upper-middle-lower” pattern between the dorsal midline and subdorsal line. The upper and lower pairs are close to the subdorsal line, while the middle pair is near the dorsal midline. Each verruca bears black setae, and in older larvae, near-circular white spots around the verrucae become clearly visible (Figure 2j). From the side view, each abdominal segment from the first to the eighth has a pair of oval spiracles (Figure 2k). There are four pairs of prolegs, each bearing biordinal crochets (Figure 2l). The anal plate is raised into a peak with three long spots, orange in the middle and white at the edges. The anal shield is oval and has six verrucae along its edge, each bearing setae. The middle pair of setae are longer. In older larvae, near-circular white spots around the verrucae become visible (Figure 2m). There is one pair of anal legs.

#### 3.1.3. Pupa

**Figure 3:**
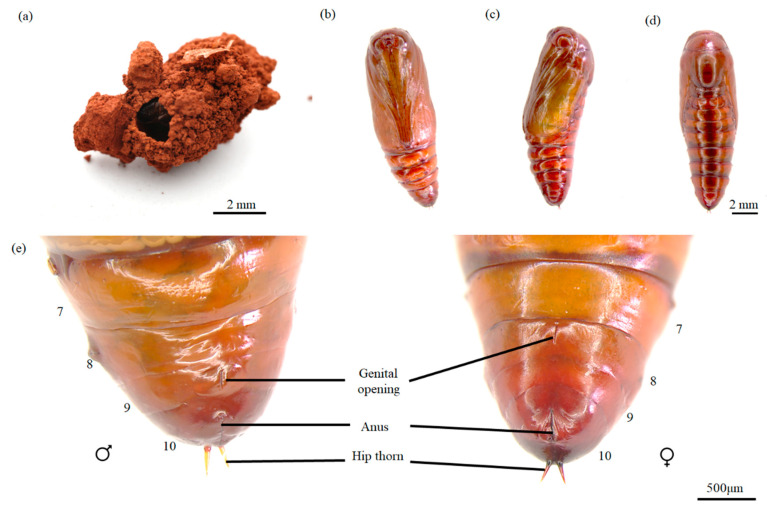
Pupal chamber and pupae of *Condica capensis*. (**a**) Pupal chamber; (**b**) pupa, dorsal view; (**c**) pupa, lateral view; (**d**) pupa, ventral view; (**e**) micrographs of male (left) and female (right) pupae, showing ventral sides of abdominal segments. Posterior abdominal segments from the 7th to the 10th are shown. The locations of the genital opening, anus, and hip thorn are indicated for each pupa.

The pupa is brown and obtect, with a smooth surface and no setae. Each abdominal segment from the second to the eighth has a pair of oval spiracles, although the spiracle on the eighth segment is reduced to a fine trace. The abdomen has a pair of V-shaped hip thorns (Figure 3b–e). There is no difference in color or appearance between male and female pupae, but the genital opening of the male pupa is located on the ninth abdominal segment, while that of the female pupa is on the eighth abdominal segment (Figure 3e).

#### 3.1.4. Adult

**Figure 4:**
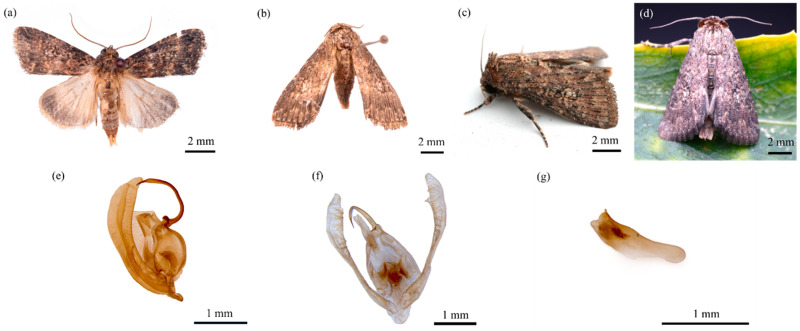
Morphological characteristics of *Condica capensis* adults. (**a**) Adult female; (**b**) adult male; (**c**) left lateral view of the adult female; (**d**) dorsal view of the adult female; (**e**) male genitalia, lateral view; (**f**) male genitalia, frontal view; (**g**) phallus.

The head is grayish brown with yellowish filiform antennae and prominent dark brown compound eyes. The thorax is covered in gray–brown scales (Figure 4a,b). The forewings are grayish brown with indistinct transverse lines. The inner line is black, with a yellowish gray inner side and an irregular, saw-tooth-shaped outer margin. The orbicular spot is grayish brown with a black border and elliptical shape. The reniform spot is yellowish gray with a dark brown ring in the center, a yellowish gray jagged outer margin, and a row of white spots below it. The subterminal fascia is slightly black, and the terminal fascia is black (Figure 4c,d). The hindwings are yellowish white with brown tips. The legs alternate between black and brown, with two spines on the hind tibia. The abdomen is yellowish-brown.

#### 3.1.5. Male Genitalia

The male genitalia are generally pale yellow. The gnathos is slender and pointed at the tip. The upper part of the tegumen is narrower than the lower part, with elongated bristles on the inner side. The harpago is slightly curved, resembling a scimitar, and bears bristles. The phallobase is elongated and located within the genital chamber (Figure 4e–g).

### 3.2. Molecular Identification

The sequencing results indicated that the sequence (PQ613989.1) has a base pair length of 658 bp, with the following base composition: A = 31.003%, T = 39.666%, C = 15.198%, and G = 14.134%. The average A + T content is 70.669%, and the average C + G content is 29.331%, showing a notable A + T bias, which is characteristic of the mitochondrial COI gene in insects. A BLAST search in NCBI revealed that the sequence shares 100% homology with the sequence of *Condica capensis* (Lepidoptera: Noctuidae), GenBank accession number NC_062101.1.

### 3.3. Phylogenetic Analysis

**Figure 5:**
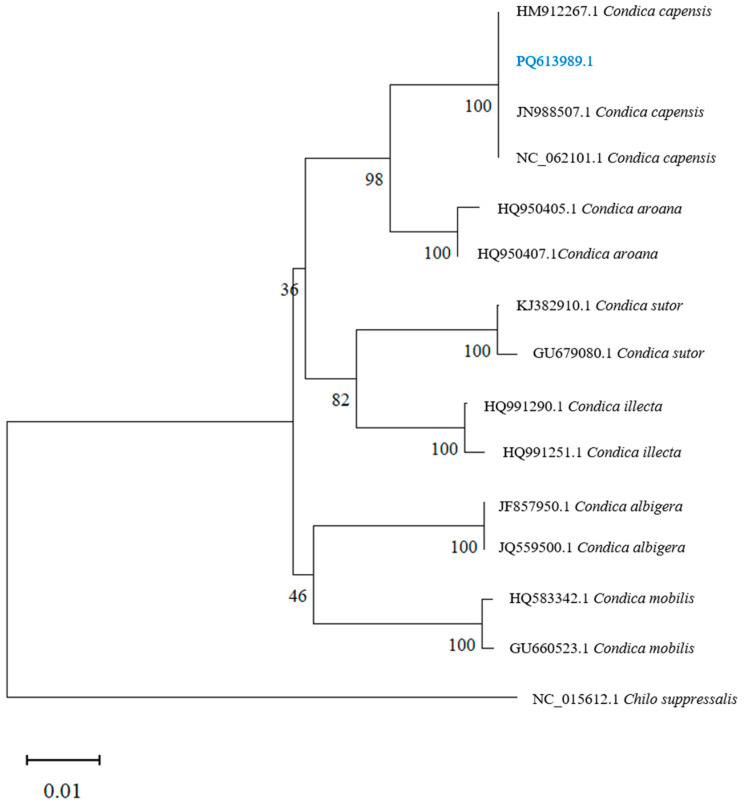
Phylogenetic tree of *Condica capensis* based on COI gene sequences using the Neighbor-Joining method.

**Figure 6:**
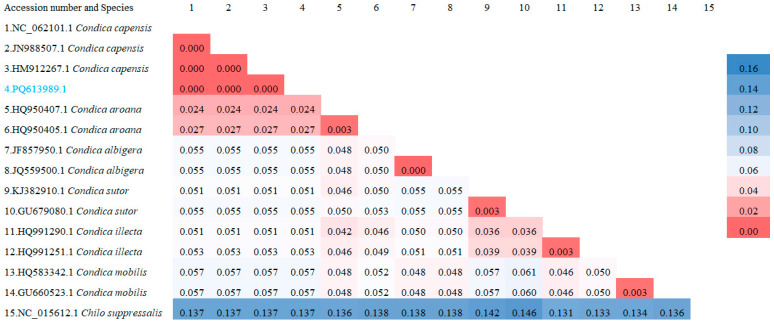
Pairwise genetic distances using the COI gene among *Condica capensis* and related species, including the out-group.

As shown in Figure 5, the phylogenetic analysis revealed that the sequence (PQ613989.1) clustered with sequences of *Condica capensis* from Canada (HM912267.1), Pakistan (JN988507.1), and Guangdong, China (NC_062101.1), with a genetic distance of 0.000, indicating no significant differentiation among populations from Canada, Pakistan, Guangdong, and Yunnan, China. Regarding interspecific differences within the genus *Condica* (Figure 6), *C. mobilis* and *C. albigera* showed greater genetic distances from *C. capensis*, with distances of 0.057 and 0.055, respectively. The closest species to *C. capensis* is *C. aroana* (HQ950407.1), with a genetic distance of 0.024.

### 3.4. Biology of Condica capensis in Safflower

#### 3.4.1. Life Cycle

The life cycle of *Condica capensis* is detailed in Table 1. The eggs of *C. capensis* hatch after an average of 3.92 ± 0.28 days. Newly hatched larvae are highly active and crawl quickly. Upon hatching, they often climb to higher places, suspend themselves with silk, search for food, feed, and then suspend themselves again with silk on leaves after feeding. First-instar larvae do not feed on the eggshells, and starvation for two days results in high mortality. Older larvae show reduced mobility, are less active, and do not spin silk. During the voracious feeding stage, larvae rest on the leaves when not feeding. Younger larvae primarily feed on tender safflower leaves, but from the third instar onward, they also feed on safflower stems and buds during the voracious feeding stage. No cannibalism was observed among larvae during food shortages, but mature larvae sometimes feed on pupae. There are six larval instars, with the first instar lasting 4.03 ± 0.18 days, the second 2.02 ± 0.34 days, the third 2.55 ± 0.57 days, the fourth 2.90 ± 1.04 days, the fifth 3.72 ± 1.37 days, and the sixth 6.42 ± 1.68 days. After the sixth instar, mature larvae burrow into the soil to a depth of 1–2 cm, secrete mucus with their mouthparts to construct an oval pupal chamber (Figure 3a), turn green, and pupate. The pupal stage lasts for 9.63 ± 1.54 days. After pupation, adults emerge, with males living for 13.17 ± 3.83 days and females for 14.33 ± 3.17 days.

#### 3.4.2. Morphometrics of *Condica capensis*

Eggs: During the rearing process, mated and unmated females were separated, and it was found that unmated females could also lay eggs, but these eggs did not hatch, indicating that parthenogenesis does not occur in females.

**Figure 7:**
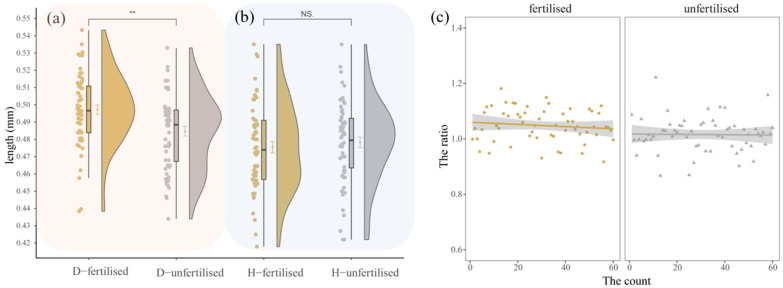
Comparison of morphological differences between fertilized and unfertilized eggs of *Condica capensis*. (**a**) Violin plot of egg diameter; (**b**) violin plot of egg height; (**c**) distribution of the ratio of egg diameter to height. Yellow indicates fertilized eggs and gray indicates unfertilized eggs. The value bars with “NS” indicate no significant difference, while asterisks denote statistically significant differences (** *p* < 0.01).

Comparison of fertilized and unfertilized eggs: As shown in Figure 7a,b, the diameter and height of fertilized eggs were 0.4972 ± 0.0209 mm and 0.4755 ± 0.0254 mm, respectively. For unfertilized eggs, the diameter and height were 0.4847 ± 0.0216 mm and 0.4783 ± 0.0236 mm, respectively. Statistical analysis revealed a significant difference in the diameter between fertilized and unfertilized eggs (t = 3.2269, df = 117.89, *p*-value = 0.00162), but no significant difference in egg height.

Egg diameter to height ratio: As shown in Figure 7c, the ratio of diameter to height for both fertilized and unfertilized eggs is approximately 1. However, the diameter of fertilized eggs is slightly larger than that of unfertilized eggs, making fertilized eggs appear flatter.

**Figure 8:**
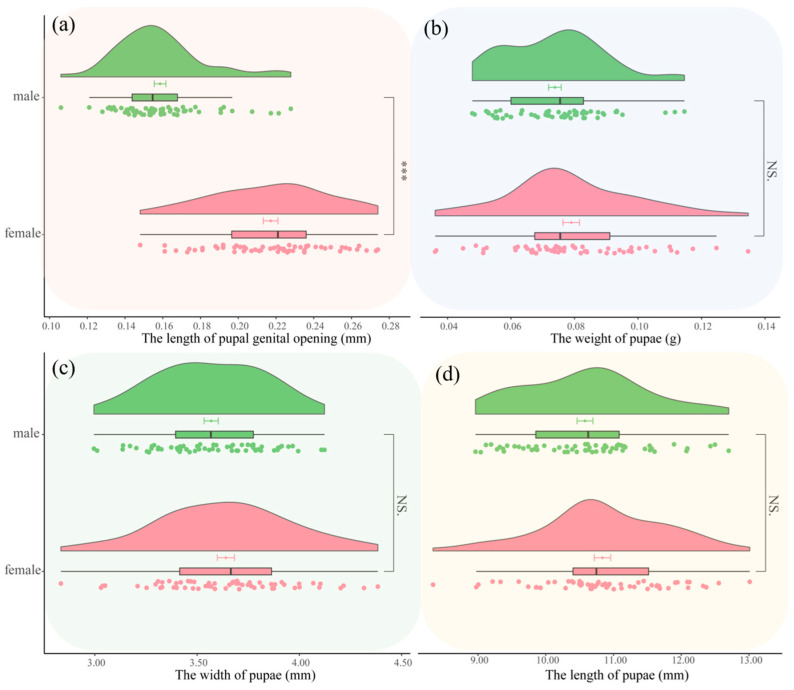
Comparison of morphological differences between male and female pupae of *Condica capensis*. (**a**) Violin plot of genital pore length; (**b**) violin plot of pupal weight; (**c**) violin plot of pupal width; (**d**) violin plot of pupal length. Green indicates male pupae, and red indicates female pupae. The value bars with “NS” indicate no significant difference, while asterisks denote statistically significant differences (*** *p* < 0.001).

Pupae: the length, width, and weight of male pupae were 10.5796 ± 0.9067 mm, 3.5687 ± 0.2695 mm, and 0.0738 ± 0.0155 g, respectively; the length of the genital pore was 0.1586 ± 0.0238 mm. For female pupae, the length, width, and weight were 10.8384 ± 0.9365 mm, 3.6405 ± 0.3256 mm, and 0.0790 ± 0.0202 g, respectively; the length of the genital pore was 0.2171 ± 0.0302 mm. Figure 8 shows no significant differences in length, width, and weight between male and female pupae, but there was a highly significant difference in the length of the genital pore (t = 11.789, df = 111.99, *p*-value < 0.001).

**Figure 9:**
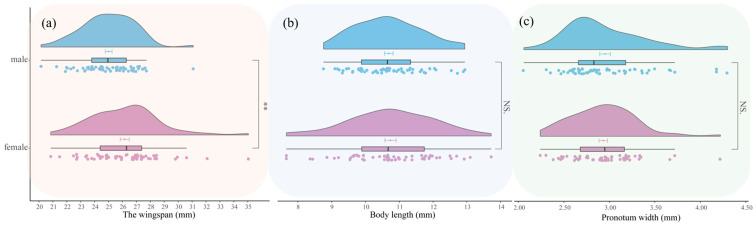
Comparison of morphological differences between male and female adults of *Condica capensis*. (**a**) Violin plot of wingspan; (**b**) violin plot of body length; (**c**) violin plot of pronotum width. Blue indicates male adults and purple indicates female adults. The value bars with “NS” indicate no significant difference, while asterisks denote statistically significant differences (** *p* < 0.01).

Adults: The wingspan, body length, and pronotum width of male adults were 25.00 ± 1.91 mm, 10.69 ± 1.00 mm, and 2.95 ± 0.46 mm, respectively. For female adults, the wingspan, body length, and body width were 26.16 ± 2.50 mm, 10.74 ± 1.28 mm, and 2.93 ± 0.36 mm, respectively. As shown in Figure 9, there were no significant differences in pronotum width and body length between male and female adults. However, the wingspan of female adults was significantly different from that of male adults (t = 2.8483, df = 110.35, *p*-value = 0.005244).

#### 3.4.3. Instar Division

**Figure 10:**
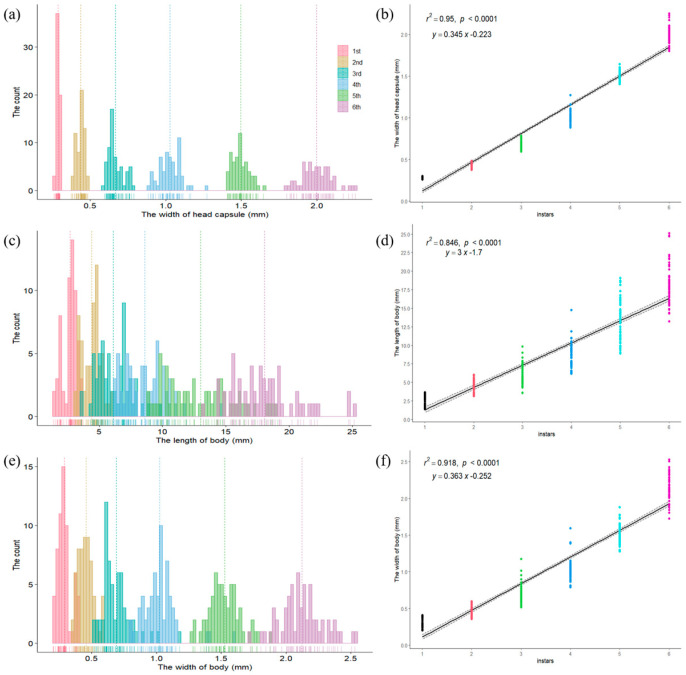
Frequency distribution and regressive relationships of *Condica capensis* larvae. (**a**) Frequency distribution of head capsule width; (**b**) regressive relationship between head capsule width and larval instar; (**c**) frequency distribution of body length; (**d**) regressive relationship between body length and larval instar; (**e**) frequency distribution of body width; (**f**) regressive relationship between body width and larval instar.

Based on the head capsule width data of *C. capensis* larvae, a frequency distribution chart was created (Figure 10). The results showed six distinct concentration areas in the frequency distribution of head capsule widths, which, according to Dyar’s rule, preliminarily indicates that *C. capensis* larvae can be divided into six instars. The head capsule width ranges for instars 1, 2, 3, 4, 5, and 6 are 0.2600–0.3000 mm, 0.3740–0.4840 mm, 0.5940–0.7870 mm, 0.8850–1.2730 mm, 1.4060–1.6450 mm, and 1.8010–2.2540 mm, respectively, with no overlap between values of different instars. The average head capsule widths for each instar were 0.2870 ± 0.0087 mm, 0.4363 ± 0.0260 mm, 0.6662 ± 0.0495 mm, 1.0270 ± 0.0711 mm, 1.4942 ± 0.0571 mm, and 1.9956 ± 0.0989 mm, respectively, with significant differences between instars (*p* < 0.05). The rationality of the instar division was tested using Dyar’s rule, Brooks index, and Crosby index, and the results showed Crosby index values for head capsule width were all less than 0.1 (Table 2), and the coefficients of variation (cv) within each instar were small. Further regression analysis of head capsule width (*y*) against instar (*x*) showed a strong linear relationship, with a regression equation of *y* = 0.345*x* − 0.223 and a correlation coefficient (R^2^) of 0.95, indicating a high correlation between head capsule width and instar. Thus, dividing *C. capensis* larvae into six instars based on head capsule width is highly reasonable.

Frequency analysis and regression analysis of body length and body width data for *C. capensis* larvae showed no distinct concentration areas in frequency distribution. The body length ranges for larvae corresponding to six instars based on head capsule width are 1.3770–3.6670 mm, 3.1410–6.0270 mm, 3.5920–9.8510 mm, 6.1490–14.7500 mm, 8.9060–19.0730 mm, and 13.2260–25.1350 mm, with body width ranges of 0.2020–0.4090 mm, 0.3590–0.5970 mm, 0.5160–1.1770 mm, 0.7910–1.5920 mm, 1.2800–1.8800 mm, and 1.7260–2.5310 mm, respectively, showing significant overlap between values of different instars. Despite the Crosby index for both body length and width being less than 0.1 (Table 2), high variability within each instar and substantial overlap between instars suggest that body length and width are not suitable for determining the instar division of *C. capensis* larvae.

#### 3.4.4. Reproductive Behavior

A total of 30 pairs of adults were observed (Table 3). Eighteen pairs successfully mated and laid eggs, while twelve pairs did not mate but still laid eggs, resulting in a mating success rate of 60%. Females laid eggs regardless of mating status. The average pre-copulation period for females was 1.61 ± 0.78 days. The pre-oviposition period for mated females was 3.22 ± 1.22 days, while that for unmated females was 5.00 ± 1.35 days, showing a significant difference (t = −3.6784, df = 21.972, *p*-value = 0.001319). Mating primarily occurred between 1 a.m. and 5 a.m. The total number of eggs laid by mated and unmated females was 364.44 ± 216.30 and 309.42 ± 164.30, respectively, with no significant difference (t = 0.79025, df = 27.419, *p*-value = 0.4362).

### 3.5. Natural Enemies of C. capensis in Safflower

**Figure 11:**
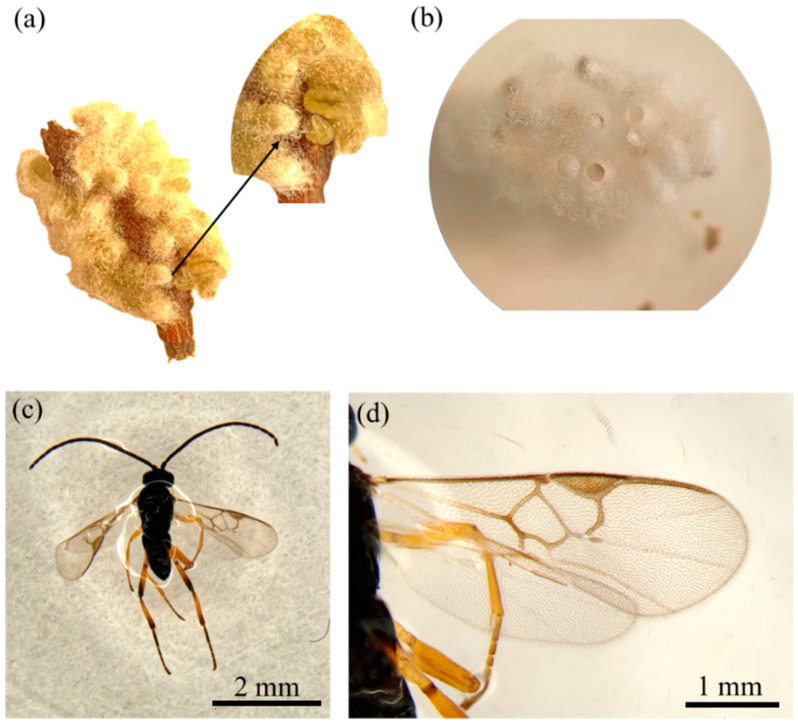
Characteristics of the parasitoid of *Condica capensis*, *Cotesia* sp. (**a**) Parasitoid larvae emerging from a *C. capensis* larva before pupating; (**b**) pupae; (**c**) adult parasitoid, dorsal view; (**d**) adult parasitoid, dorsal view of right wings.

Approximately one week after collecting *C. capensis* larvae from the field and rearing them indoors, it was observed that the larvae’s bodies gradually shrank, and parasitoid larvae began emerging from the host larvae, pupating shortly afterward (Figure 11a). About ten days later, emerged adult parasitoids were observed (Figure 11b). These parasitoids were identified by Professor Wang Xiaoyi as belonging to the genus *Cotesia* (Figure 11c,d), indicating that *Cotesia* sp. parasitoids are capable of parasitizing *C. capensis*.

## 4. Discussion

The AR6 Synthesis Report: Climate Change 2023 highlights significant climate changes, including rising temperatures, sea level rise, altered precipitation patterns, and increased frequency and intensity of extreme weather events [24]. These climatic shifts are altering existing ecological niches and creating new ones, influencing pest distribution [25]. Originally native to Africa, *C. capensis* has recently been recorded in Europe, North America, Central Asia, Southeast Asia, and Australia [15]. Yunnan Province, located in the southwestern plateau of China, features a diverse climate and topography that create favorable conditions for invasive species. The discovery of *C. capensis* in Yunnan is concerning, and increased monitoring is necessary to prevent its spread to other provinces in China.

The genus *Condica* comprises around 77 medium-sized moth species, though the exact number is uncertain due to taxonomic revisions, including species from the genus *Perigea* [14]. The genus is most diverse in tropical regions, particularly the Neotropics [26]. In China, six species of *Condica* have been recorded, including *C. cyclica*, *C. illustrata*, *C. illecta*, *C. albigutta*, *C. dolorosa*, and *C. capensis*. Except for *C. illustrata*, the other five species were originally classified under *Perigea* [27]. The Noctuidae family, the largest in Lepidoptera [28], includes many economically significant pests, such as *Agrotis ipsilon*, *Helicoverpa armigera*, *Spodoptera frugiperda*, *Mythimna separata*, and *Spodoptera litura* [29,30,31,32,33]. Among these, *S. frugiperda* is a major migratory pest, recognized by FAO for its global threat to crops [34]. The simultaneous occurrence of multiple noctuid pests complicates identification, making morphological traits essential for rapid and accurate pest diagnosis.

Insect taxonomy is fundamental to entomological research, but the vast number of species makes traditional identification methods challenging. The advent of DNA barcoding in 2003 revolutionized species identification. Hebert demonstrated the feasibility of using mitochondrial COI gene sequences for identifying species, successfully identifying 200 closely related Lepidoptera species [35]. Ahmad used COI barcoding to identify *Chilo partellus*, a new pest in Pakistan [36], and Abdel-Galil used COI to accurately identify *Deudorix livia* in Egypt [37]. DNA barcoding is a rapid, cost-effective, and accurate method for species identification.

Measuring morphological metrics is also critical for insect taxonomy. Studies show a correlation between flight ability and body size, including wingspan, thorax, and abdomen dimensions [38,39]. For example, Galaschi-Teixeira used geometric morphometrics to distinguish 11 species of *Melipona* based on wing morphology [40].

While some noctuid larvae feed on egg chorion when food is scarce [41], no such behavior was observed in *C. capensis*. Cannibalism is commonly observed in noctuid larvae such as *S. frugiperda*, *S. exigua*, and *H. armigera* [41,42,43], but no cannibalism was noted in *C. capensis*, although older larvae fed on pupae when food was limited. Like many noctuids, *C. capensis* exhibits nocturnal mating, courtship, and oviposition behaviors [44]. Unlike *S. frugiperda* and *Mamestra configurata*, which lay eggs in clusters, *C. capensis* lays eggs singly [45,46].

FAO estimates that pests cause up to 40% of global crop losses annually, resulting in economic damages exceeding 220 billion dollars, with invasive insects contributing at least 70 billion dollars [47]. The prolonged use of chemical pesticides leads to ecological harm and is unsustainable. Laboratory rearing of *C. capensis* revealed its voracious feeding habits, which significantly reduce safflower yields. Thus, ecologically safe and effective control measures are needed. Natural parasitoids, such as *Cotesia* spp., have been found to parasitize *C. capensis*. Studies show that *Cotesia* species are effective parasitoids of various lepidopteran pests, including *S. exigua*, *Diatraea saccharalis*, *Plutella xylostella*, and *Pieris rapae* [48,49,50,51]. Releasing *Cotesia* parasitoids to control *C. capensis* could be a sustainable, environmentally friendly alternative to chemical pesticides, promoting ecosystem health.

## 5. Conclusions

We report for the first time that *Condica capensis* affects safflower in Yunnan Province. This study examines its morphology, lifecycle, and reproductive behavior to aid in better identification and management. While DNA barcoding serves as a quick and reliable method for detecting pests, traditional identification methods remain important. Using parasitoid wasps (*Cotesia* sp.) as natural enemies instead of chemical pesticides is a promising and sustainable approach. Regular monitoring of this pest and its natural enemies is crucial to avoid economic and environmental damage. This discovery highlights the importance of monitoring and management strategies to prevent the spread of *C. capensis* in China and worldwide.

## Figures and Tables

**Table 1:** Life cycle parameters of *Condica capensis* on *Carthamus tinctorius*.

Life Stages		Mean ± SE (days)	Variation (days)
Eggs period		3.92 ± 0.28	3.00–4.00
Larval period	1st	4.03 ± 0.18	4.00–5.00
	2nd	2.02 ± 0.34	1.00–3.00
	3rd	2.55 ± 0.57	1.00–3.00
	4th	2.90 ± 1.04	1.00–5.00
	5th	3.72 ± 1.37	2.00–10.00
	6th	6.42 ± 1.68	4.00–14.00
Pupal period		9.63 ± 1.54	6.00–14.00
Male		13.17 ± 3.83	4.00–20.00
Female		14.33 ± 3.17	5.00–20.00

**Table 2:** Measurements and statistics of three morphological variables used to determine the instars of *Condica capensis*.

Variable	Instars	Mean ± SE (mm)	Variation (mm)	Coefficient of Variance (%)	Brooks Index	Crosby Index
Head capsule width	1	0.2870 ± 0.0087 f	0.2600–0.3000	3.0161		
	2	0.4363 ± 0.0260 e	0.3740–0.4840	5.9598	1.5199	
	3	0.6662 ± 0.0495 d	0.5940–0.7870	7.4241	1.5271	0.0048
	4	1.0270 ± 0.0711 c	0.8850–1.2730	6.9240	1.5416	0.0095
	5	1.4942 ± 0.0571 b	1.4060–1.6450	3.8230	1.4549	−0.0562
	6	1.9956 ± 0.0989 a	1.8010–2.2540	4.9547	1.3355	−0.0821
Body Width	1	0.2903 ± 0.0515 f	0.2020–0.4090	17.7495		
	2	0.4570 ± 0.0610 e	0.3590–0.5970	13.3539	1.5742	
	3	0.6902 ± 0.1191 d	0.5160–1.1770	17.2499	1.5103	−0.0406
	4	1.0265 ± 0.1343 c	0.7910–1.5920	13.0806	1.4872	−0.0153
	5	1.5257 ± 0.1245 b	1.2800–1.8800	8.1585	1.4863	−0.0006
	6	2.1234 ± 0.1673 a	1.7260–2.5310	7.8810	1.3918	−0.0636
Body Length	1	2.6906 ± 0.5643 f	1.3770–3.6670	20.9745		
	2	4.4065 ± 0.7545 e	3.1410–6.0270	17.1232	1.6378	
	3	6.1109 ± 1.3043 d	3.5920–9.8510	21.3444	1.3868	−0.1532
	4	8.6005 ± 1.6687 c	6.1490–14.7500	19.4025	1.4074	0.0149
	5	13.0174 ± 2.7303 b	8.9060–19.0730	20.9745	1.5136	0.0754
	6	18.0580 ± 2.6158 a	13.2260–25.1350	14.4854	1.3872	−0.0835

Note: Data within the same column and for the same measured parameter followed by different lowercase letters indicate significant differences at *p* < 0.05 (One-way ANOVA, Duncan’s test).

**Table 3:** Oviposition behavior of female *Condica capensis* under different treatments.

Treatment	Number of Observations (ind.)	Pre-Copulation Period (days)		Pre-Oviposition Period (days)		Number of Eggs (Grains)	
		Mean ± SE	Variation	Mean ± SE	Variation	Mean ± SE	Variation
Mated female	18	1.61 ± 0.78	0.00–3.00	3.22 ± 1.22 b	1.00–6.00	364.44 ± 216.30 a	36.00–913.00
Virgin female	12	/	/	5.00 ± 1.35 a	3.00–7.00	309.42 ± 164.30 a	88.00–662.00

Note: Data within the same column and for the same measured parameter followed by different lowercase letters indicate significant differences at *p* < 0.01 (*t*-test).

## Data Availability

The data supporting the results are available in a public repository at: https://figshare.com/s/0ea96ecb0f7840836a84, accessed on 15 January 2025.

## References

[1] Baek S.C., Yi S.A., Lee B.S., Yu J.S., Kim J.-C., Pang C., Jang T.S., Lee J., Kim K.H. (2021). Anti-Adipogenic Polyacetylene Glycosides from the Florets of Safflower (*Carthamus tinctorius*). Biomedicines.

[2] Yao D., Wang Z., Miao L., Wang L. (2016). Effects of Extracts and Isolated Compounds from Safflower on Some Index of Promoting Blood Circulation and Regulating Menstruation. J. Ethnopharmacol..

[3] Joseph R.S. (1996). Safflower.

[4] Jin J., Liu J., Wang D., Gao S., Zhao F., Wang Y. (2022). Reconstruction of Traditional Safflower (*Carthamus tinctorius* L.) Dyeing and Red Colors in the Qing Dynasty (17th–19th Century). Dye Pigment..

[5] Pearl S.A., Burke J.M. (2014). Genetic Diversity in *Carthamus tinctorius* (Asteraceae; Safflower), an Underutilized Oilseed Crop. Am. J. Bot..

[6] Kammili A., Yadav P. (2022). Enhancing Oleic Acid and Oil Content in Low Oil and Oleic Type Indian Safflower (*Carthamus tinctorius* L.). Ind. Crops Prod..

[7] Khalid N., Khan R.S., Hussain M.I., Farooq M., Ahmad A., Ahmed I. (2017). A Comprehensive Characterisation of Safflower Oil for Its Potential Applications as a Bioactive Food Ingredient—A Review. Trends. Food. Sci. Technol..

[8] Chapman M.A., Hvala J., Strever J., Burke J.M. (2010). Population Genetic Analysis of Safflower (*Carthamus tinctorius*; Asteraceae) Reveals a near Eastern Origin and Five Centers of Diversity. Am. J. Bot..

[9] Wu X., Cai X., Ai J., Zhang C., Liu N., Gao W. (2021). Extraction, Structures, Bioactivities and Structure-Function Analysis of the Polysaccharides from Safflower (*Carthamus tinctorius* L.). Front. Pharmacol..

[10] Saeidi K., Azura A.N., Omar D., Abood F. (2012). Pests of Safflower (*Carthamus tinctorious* L.) and Their Natural Enemies in Gachsara, Iran. South Asian J. Exp. Biol..

[11] Androcioli H.G., Hoshino A.T., Pastório M.A., Cardoso P.C., de Araújo P.M., Fernandes T.A.P., Menezes A.O. (2017). First Record of Euphoria Lurida Fabricius (Coleoptera: Scarabaeidae) Injurious to Safflower (*Carthamus tinctorius* L.) (Asterales: Asteraceae) in Brazil. Neotrop. Entomol..

[12] Yucel C., Ozdemir I., Coral D. (2022). DNA Barcoding Data of Aphids (Hemiptera: Aphidomorpha) in Safflower *(Carthamus tinctorius* L.) with New Host Plant Records in Turkey. J. Entomol. Res. Soc..

[13] *Condica capensis* in Catalogue of Life China: 2024 Annual Checklist, Beijing, China. http://www.sp2000.org.cn/species/show_species_details/1CE8B40F-2F79-4EC9-93A4-059E5349ED80.

[14] Rijllo G., La Cava S., Zucco G., Scalercio S. (2024). Gone with the Wind? *Condica capensis* (Guenée 1852), a Migrant Species New for Italy (Lepidoptera: Noctuidae). Nat. Hist. Sci..

[15] Amer A. (2020). Revision of Family Noctuidae (4) Subfamilies “Bryophilinae, Condicinae, Cuculliinae, Eriopinae and Eustrotiinae” of Egypt (Lepidoptera, Noctuidae). Egypt. Acad. J. Biol. Sci. A Entomol..

[16] Rangarajan A.V., Mahadevan N.R., Iyemperumal S. (1977). Pest Complex of Sunflower (*Helianthus annus* Linn.) in Tamil Nadu. Indian J. Entomol..

[17] Balikai R.A. (2000). Insect Pests of Safflower and Their Natural Enemies in Northern Karnataka. Karnataka J. Agric. Sci..

[18] Kranthi S., Kranthi K.R., Rishi K., Udikeri S.S., Rao G.M.V.P., Zanwar P.R., Nagrare V.N., Naik C.B., Singh V., Ramamurthy V.V. (2011). Emerging and key insect pests on Bt cotton-their identification, taxonomy, genetic diversity and management. Proceedings of the World Cotton Research Conference-5.

[19] Chen L., Pan Q., Waqas M.S., Liu T. (2020). Morphological Traits for Sex Identification of the Oriental Armyworm, *Mythimna separata* (Lepidoptera: Noctuidae). J. Integr. Agric..

[20] Folmer O., Black M., Hoeh W., Lutz R., Vrijenhoek R. (1994). DNA Primers for Amplification of Mitochondrial Cytochrome c Oxidase Subunit I from Diverse Metazoan Invertebrates. Mol. Mar. Biol. Biotechnol..

[21] Tamura K., Stecher G., Kumar S. (2021). MEGA11 Molecular Evolutionary Genetics Analysis Version 11. Mol. Biol. Evol..

[22] Saitou N., Nei M. (1987). The Neighbor-Joining Method: A New Method for Reconstructing Phylogenetic Trees. Mol. Biol. Evol..

[23] Loerch C.R., Cameron E.A. (1983). Determination of Larval Instars of the Bronze Birch Borer, *Agrilus anxius* (Coleoptera: Buprestidae)1. Ann. Entomol. Soc. Am..

[24] AR6 Synthesis Report: Climate Change 2023—IPCC. https://www.ipcc.ch/report/sixth-assessment-report-cycle/.

[25] Sinelnikov I.G., Siedhoff N.E., Chulkin A.M., Zorov I.N., Schwaneberg U., Davari M.D., Sinitsyna O.A., Shcherbakova L.A., Sinitsyn A.P., Rozhkova A.M. (2021). Expression and Refolding of the Plant Chitinase from Drosera Capensis for Applications as a Sustainable and Integrated Pest Management. Front. Bioeng. Biotechnol..

[26] Walker F. (1856). List of the Specimens of Lepidopterous Insects in the Collection of the British Museum.

[27] Browse Taxonomic Tree in Catalogue of Life China: 2024 Annual Checklist, Beijing, China. http://www.sp2000.org.cn/browse/browse_this_taxa/ee2b3e1d-5eaf-4841-a579-eb580c239491.

[28] Hussain Z., Sarwar Z.M., Akbar A., Alhag S.K., Ahmed N., Alam P., Almadiy A.A., Zouidi F., Jawalkar N.B. (2022). Spatiotemporal Distribution Patterns of Pest Species (Lepidoptera: Noctuidae) Affected by Meteorological Factors in an Agroecosystem. Agriculture.

[29] Liu Y., Fu X., Feng H., Liu Z., Wu K. (2015). Trans-Regional Migration of *Agrotis ipsilon* (Lepidoptera: Noctuidae) in North-East Asia. Ann. Entomol. Soc. Am..

[30] Zhou Y., Wu Q., Zhao S., Guo J., Wyckhuys K.A.G., Wu K. (2019). Migratory *Helicoverpa armigera* (Lepidoptera: Noctuidae) Exhibits Marked Seasonal Variation in Morphology and Fitness. Environ. Entomol..

[31] Zhang X.-Y., Huang L., Liu J., Zhang H.-B., Qiu K., Lu F., Hu G. (2023). Migration Dynamics of Fall Armyworm *Spodoptera frugiperda* (Smith) in the Yangtze River Delta. Insects.

[32] Cang X., Zhao S., Yang X., Yuan H., Liu J., Liu D., Yang X., Wu K. (2023). Migration Monitoring and Route Analysis of the Oriental Armyworm *Mythimna separata* (Walker) in Northeast China. Agriculture.

[33] Song Y., Cang X., He W., Zhang H., Wu K. (2024). Migration Activity of *Spodoptera litura* (Lepidoptera: Noctuidae) between China and the South-Southeast Asian Region. Inscets.

[34] Wang X., Du Z., Duan Y., Liu S., Liu J., Li B., Ma L., Wu Y., Tian L., Song F. (2024). Population Genomics Analyses Reveal the Role of Hybridization in the Rapid Invasion of Fall Armyworm. J. Adv. Res..

[35] Hebert P.D.N., Cywinska A., Ball S.L., deWaard J.R. (2003). Biological Identifications through DNA Barcodes. Proc. R. Soc. Lond. Ser. B Biol. Sci..

[36] Ahmad S., Idrees A., Haider M.U., Rasool B., Mehmood R., Afzal A., Javaid A., Shad N., Ashrafl S., Liz J. (2022). COI-Gene Based Molecular Identification of *Chilo partellus* Swinhoe (Lepidoptera: Pyralidae) Infesting Maize in Lahore, Pakistan. Philipp. Agric. Sci..

[37] Abdel-Galil F.A., Mousa S.E., Abou-Elhagag G.H., Ahmed A.M.M., Al-Farga A., Allam M., Mahmoud M.A.B. (2023). Morphogenetic Identification of a New Record Deudorix Livia (Lepidoptera: Lycaenidae) in Assiut Governorate of Northern Upper Egypt. Sci. Rep..

[38] Sekar S. (2012). A Meta-Analysis of the Traits Affecting Dispersal Ability in Butterflies: Can Wingspan Be Used as a Proxy?. J. Anim. Ecol..

[39] Perrard A. (2020). Wasp Waist and Flight: Convergent Evolution in Wasps Reveals a Link between Wings and Body Shapes. Am. Nat..

[40] Galaschi-Teixeira J.S., Veiga J.C., Tavares V.D.C., Imperatriz-Fonseca V.L. (2024). Geometric Morphometrics of the Wings of Amazonian Species of *Melipona* (Illiger, 1806) (Hymenoptera: Apidae). Zootaxa.

[41] Queiroz-Santos L., Casagrande M.M., Specht A. (2018). Morphological Characterization of *Helicoverpa armigera* (Hubner) (Lepidoptera: Noctuidae: Heliothinae). Neotrop. Entomol..

[42] Chapman J.W., Williams T., Escribano A., Caballero P., Cave R.D., Goulson D. (1999). Age-Related Cannibalism and Horizontal Transmission of a Nuclear Polyhedrosis Virus in Larval *Spodoptera frugiperda*. Ecol. Entomol..

[43] Elvira S., Williams T., Caballero P. (2010). Juvenile Hormone Analog Technology: Effects on Larval Cannibalism and the Production of Spodoptera Exigua (Lepidoptera: Noctuidae) Nucleopolyhedrovirus. J. Econ. Entomol..

[44] Groot A.T. (2014). Circadian Rhythms of Sexual Activities in Moths: A Review. Front. Ecol. Evol..

[45] Ulmer B., Gillott C., Erlandson M. (2003). Conspecific Eggs and Bertha Armyworm, *Mamestra configurata* (Lepidoptera: Noctuidae), Oviposition Site Selection. Environ. Entomol..

[46] Volp T.M., Zalucki M.P., Furlong M.J. (2022). What Defines a Host? Oviposition Behavior and Larval Performance of *Spodoptera frugiperda* (Lepidoptera: Noctuidae) on Five Putative Host Plants. J. Econ. Entomol..

[47] Jin M., North H.L., Peng Y., Liu H., Liu B., Pan R., Zhou Y., Zheng W., Liu K., Yang B. (2023). Adaptive Evolution to the Natural and Anthropogenic Environment in a Global Invasive Crop Pest, the Cotton Bollworm. Innov..

[48] Hervet V.A.D., Murillo H., Fernández-Triana J.L., Shaw M.R., Laird R.A., Floate K.D. (2014). First Report of *Cotesia vanessae* (Hymenoptera: Braconidae) in North America. Can. Entomol..

[49] Matioli T.F., Zanardi O.Z., Yamamoto P.T. (2019). Impacts of Seven Insecticides on *Cotesia flavipes* (Cameron) (Hymenoptera: Braconidae). Ecotoxicology.

[50] Song W., Liu L., Li P., Sun H., Qin Y. (2014). Analysis of the Mating and Reproductive Traits of *Plutella xylostella* (Lepidoptera: Plutellidae). J. Insect Sci..

[51] Weis J.J., Gray H.L., Heimpel G.E. (2016). High Hyperparasitism of *Cotesia rubecula* (Hymenoptera: Braconidae) in Minnesota and Massachusetts. J. Kans. Entomol. Soc..

